# Prognostic scores based on the preoperative plasma fibrinogen and serum albumin level as a prognostic factor in patients with upper urinary tract urothelial carcinoma

**DOI:** 10.18632/oncotarget.16483

**Published:** 2017-03-22

**Authors:** Jianfeng Cui, Meng Yu, Ning Zhang, Shiyu Wang, Yaofeng Zhu, Shouzhen Chen, Kejia Zhu, Jian Du, Hongda Zhao, Xigao Liu, Pengxiang Chen, Wenbo Wang, Dongqing Zhang, Benkang Shi

**Affiliations:** ^1^ Department of Urology, Qilu Hospital of Shandong University, Jinan, P.R. China; ^2^ Department of Radiation Oncology, Qilu Hospital, Shandong University, Jinan, P.R. China; ^3^ School of Medicine, Chinese Academy of Medical Sciences and Peking Union Medical College, Beijing, P.R. China

**Keywords:** upper urinary tract urothelial carcinoma, plasma fibrinogen, serum albumin, prognosis

## Abstract

This study is to clarify the prognostic value of preoperative plasma fibrinogen and serum albumin level, as known as FA score, in a cohort of Chinese patients with upper urinary tract urothelial carcinoma (UTUC). We retrospectively evaluated clinicopathological data on 169 patients who underwent surgery between 2006 and 2013. The FA score was calculated based on cutoff values of 3.53g/L for fibrinogen and 43.56 g/L for albumin. Overall survival and cancer specific survival was assessed using the Kaplan-Meier method and the equivalences of the curves were tested by log-rank tests. The Cox proportional hazards regression model was applied in univariate and multivariate analyses. In univariate analysis, tumor size, tumor grade, pathological T stage and FA score were significantly associated with overall survival and cancer specific survival, and multivariate Cox proportional hazards regression analysis identified FA score (score 1: HR=3.486, 95%CI 1.358-8.948, *p*=0.009; HR=3.485, 95%CI 1.363-8.913, *p*=0.009, respectively; score 2: HR=5.509, 95%CI 2.144-14.158, *p*<0.001; HR=5.521, 95%CI 2.074-14.697, *p*=0.001, respectively) was an independent predictor for overall survival and cancer specific survival. The evaluation of preoperative FA score can be regarded as an independent prognostic factor for predicting overall survival and cancer specific survival in UTUC. The fibrinogen and albumin levels are low cost and easy accessibility in clinical practice.

## INTRODUCTION

Urothelial carcinomas (UCs) have become the fourth most common cancer, However, upper urinary tract urothelial cell carcinomas (UTUCs) are relatively uncommon (only about 5-10% of UCs) but aggressive malignant disease [1, 2]. In 17% of cases, concurrent bladder cancer (BCa) is present [3]. UTUCs that invade the muscle layer usually have a very poor prognosis. The 5-year cancer specific survival (CSS) is < 50% for pT2 or pT3 and < 10% for pT4 [1, 4].

According to the latest published reports [1, 5] on UTUC, pathological T stage and tumor grade are regarded as prognostic indicators of great importance, and several other prognostic parameters, such as tumor size and lymph node involvement, were suggested to predict prognosis in UTUC. Predicting the outcome of each individual patient accurately is of great concern. However, reports on the preoperative prognostic factors are still limited in UTUC. Some groups illuminated certain prognostic indexes or models to predict the clinical outcome of UTUC [6, 7]. Increasing evidence shows that hemostatic factors, nutritional deficiencies and systemic inflammatory response (SIR) might play an important role in the development and progress of human cancer [8]. Several studies show that preoperative plasma fibrinogen which plays a vital part in clot formation, independently predicts prognosis in various human cancers [9–11]. Meanwhile, hypoproteinemia reflects SIR and has been reported as an important predictor of clinical outcomes in various types of cancer [8, 12, 13]. Furthermore, serum albumin level has been regarded as a crucial parameter of malnutrition.

Previous three studies [6, 9, 14] have shown the prognostic impact of preoperative plasma fibrinogen in a cohort of Chinese, Japanese and European populations, and all three studies have proven the preoperative plasma fibrinogen level as an independent factor in patients with UTUC. Ku et al [8] noted that preoperative hypoalbuminemia was an independent predictor of poor prognosis in patients with UTUC. Matsuda et al [15] recently reported that plasma fibrinogen and serum albumin level, as known as FA score, could predict prognosis of patients with esophageal cancer for overall survival (OS) and disease-free survival. To our knowledge, the value of FA score has not used to detect in patients with UTUC. Thus, we decided to clarify the prognostic value of preoperative FA score in a cohort of Chinese patients with UTUC.

## RESULTS

### Baseline clinicopathologic characteristics

Of the 180 patients with UTUC who underwent surgery at our institution between January 2006 and December 2013, and 11 patients (6.1%) was excluded by the criteria. The mean (standard deviation, SD) follow-up duration was 53.7 (31.3) months. The clinicopathologic characteristics of 169 patients are shown in Table 1. 107 (63.3%) were male and 62 (36.7%) were female, and mean (SD) age of the study cohort was 65.66 (9.98). 38 (22.5%) patients had the history of smoking and 15 (8.9%) patients had the history of bladder cancer. In 66 (39.1%) patients the tumor was located in the renal pelvis, in 98 patients (58.0%) patients the tumor was located in the ureter and in 5 (2.9%) patients the tumor located both in renal pelvis and ureter. Multifocal tumor was found in only 10 (5.9%) patients. Tumors in 116 (68.6%) patients were less than 4 centimeter (cm). Tumor grade was G1 or G2 in 46 (27.2%) patients and G3 in 123 (72.8%). Pathological T stage was pTa/T1 in 57 (33.7%) patients and pT2-4 in 112 (66.3%). Lymph node metastasis was found in 11 (6.5%) patients. Lymphovascular invasion (LVI) was positive in 32 (18.9%) patients. And 147 (87.0%) patients were underwent radical nephroureterectomy (RNU) and 22 (13.0%) were underwent segment resection. Only 14 (8.3%) patients didn’t receive any therapy (Table 1).

**Table 1:** Clinicopathologic characteristics and FA score of 169 patients with upper tract urothelial cancer (UTUC)

		All Patients (n=169)	FA score			P value
			0 (n=36)	1 (n=79)	2 (n=54)	
Gender	Male/Female	107 (63.3%)/62 (36.7%)	19 (52.8%)/17 (47.2%)	54 (68.4%)/25 (31.6%)	34 (63.0%)/20 (37.0%)	0.274
Age, year	Mean±SD	65.66±9.98	62.90±10.71	66.00±9.70	66.94±9.74	0.166
	Median (range)	66 (36-87)	63.5 (42-85)	67 (40-84)	68 (36-87)	
History of Smoking	Yes	38(22.5%)	7 (19.4%)	22 (27.8%)	9 (16.7%)	0.28
	no	131(77.5%)	29 (80.6%)	57 (72.2%)	45 (83.3%)	
History of Bladder Cancer n(%)	Yes	15 (8.9%)	3 (8.3%)	10 (12.7%)	2 (3.7%)	0.202
	No	154 (91.1%)	33 (91.7%)	69 (87.3%)	52 (96.3%)	
Tumor Location n(%)	Renal pelvis	66 (39.1%)	18 (50.0%)	33 (41.8%)	15 (27.8%)	0.066
	Ureter	98 (58.0%)	16 (44.4%)	43 (54.4%)	39 (72.2%)	
	Both	5 (2.9%)	2 (5.6%)	3 (3.8%)	0 (0%)	
Tumor Focality n(%)	Unifocal	159 (94.1%)	34 (94.4%)	74 (93.7%)	51 (94.4%)	0.978
	Multifocal	10(5.9%)	2 (5.6%)	5 (6.3%)	3 (5.6%)	
Tumor Size n(%)	<4cm	116 (68.6%)	26 (72.2%)	49 (62.0%)	41 (75.9%)	0.207
	≥4cm	53 (31.4%)	10 (27.8%)	30 (38.0%)	13 (24.1%)	
Tumor Grade n(%)	G1-2	46 (27.2%)	13 (36.1%)	16 (20.3%)	17 (31.5%)	0.145
	G3	123 (72.8%)	23 (63.9%)	63 (79.7%)	37 (68.5%)	
Pathological T Stage n(%)	pTa/T1	57 (33.7%)	14 (38.9%)	30 (38.0%)	13 (24.1%)	0.282
	pT2-4	112 (66.3%)	22 (61.1%)	49 (62.0%)	41 (75.9%)	
Lymph node metastasis n(%)	pNx/ pN0	158 (93.5%)	36 (100%)	77 (97.4%)	45 (83.3%)	0.001
	pN+	11 (6.5%)	0 (0%)	2 (2.6%)	9 (16.7%)	
Lymphovascular invasion	Negative	137 (81.1%)	31 (86.1%)	66 (83.5%)	40 (74.1%)	0.268
	Positive	32 (18.9%)	5 (13.9%)	13 (16.5%)	14 (25.9%)	
ECOG-PS n(%)	0	159 (94.1%)	35 (97.2%)	74 (93.7%)	50 (92.6%)	0.645
	≥1	10 (5.9%)	1 (2.8%)	5 (6.3%)	4 (7.4%)	
Operation approach n(%)	RNU	147 (87.0%)	34 (94.4%)	69 (87.3%)	44 (81.5%)	0.2
	SR	22 (13.0%)	2 (5.6%)	10 (12.7%)	10 (18.5%)	
Adjuvant Chemotherapy or Radiotherapy n(%)	Yes	155 (91.7%)	33 (91.7%)	71 (89.9%)	51 (94.4%)	0.643
	No	14 (8.3%)	3 (8.3%)	8 (10.1%)	3 (5.6%)	
Albumin (g/L)	Mean±SD	41.84±4.15	46.22±2.04	41.23±4.18	39.80±2.86	
	Median (range)	42.0 (27.3-52.7)	45.8 (43.7-52.7)	41.4 (27.3-50.7)	40.0 (34.1-43.6)	
Fibrinogen (g/L)	Mean±SD	3.59±1.06	2.96±0.44	3.18±0.76	4.62±0.99	
	Median (range)	3.39 (1.50-7.70)	3.00 (2.14-3.92)	3.03 (1.50-5.17)	4.30(3.53-7.7)	

SD = Standard Deviation, pN+=pN1, pN2 or pN3, ECOG-PS = Eastern Cooperative Oncology Group performance status, RNU = Radical nephroureterectomy, SR = Segmental resection

### Distribution of preoperative fibrinogen, albumin, and FA score

The median (range) preoperative serum albumin level was 42.0g/L (27.3-52.7) and the median preoperative plasma fibrinogen level was 3.39 g/L (1.50-7.70) (Table 1).

**Figure 1:**
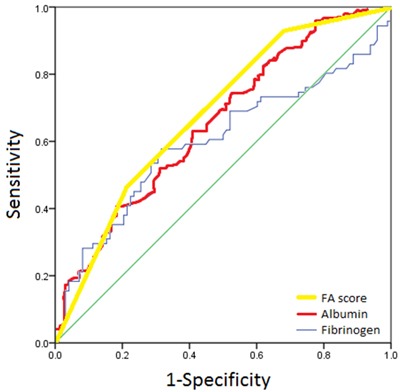
ROC analysis of optimal fibrinogen, albumin and FA score cutoff

Fibrinogen and albumin were categorized into 2 subgroups, in which the optimal cutoff value of 3.53g/L and 43.65g/L, respectively, and the patients were divided into 3 groups according to the optimal cutoff value of both fibrinogen and albumin (Figure 1). We combined the two subgroups to define the FA score as follows: patients with an elevated fibrinogen (> 3.53g/L) level and decreased serum albumin (< 43.65g/L) were assigned a score of 2, those with only one of these two abnormalities were assigned a score of 1, and those with neither of the two abnormalities were classified as having a score of 0.

### Correlation between preoperative FA score and clinicopathologic characteristics

By the threshold mentioned above, 36 (21.3%) patients had an FA score of 0, 79 (46.7 %) had an FA score of 1, and 54 (32.0 %) had a FA score of 2 (Table 1). No significant differences in gender, age, history of smoking, history of bladder cancer, tumor location, focality, size, grade, pathological T stage, LVI, Eastern Cooperative Oncology Group performance status (ECOG-PS), operation approach, adjuvant treatment were identified between the three groups. Nevertheless, lymph node metastasis was found significantly related with higher preoperative FA score (*p* = 0.001).

### Prognostic value of the preoperative FA score in predicting survival for patients with UTUC

During the follow-up period, 71 (42%) patients died of all causes and 63 (37.3%) died of cancer-specific causes, with a median OS of 47 months.

In univariate analysis, no survival difference was found in gender, age, history of smoking and LVI. Tumor size (< 4cm or ≥ 4cm), tumor grade (G1-2 or G3), pathological T stage (pTa/T1 or pT2-4) and preoperative FA score (score 0 or score 1/2) were significantly associated with OS and CSS (Table 2). To clarify the independent prognostic value of FA score for OS and CSS, multivariate Cox proportional hazards regression analysis using tumor size, tumor grade, tumor stage and preoperative FA score as covariates revealed that tumor size (< 4cm or ≥ 4cm, HR = 1.865, 95%CI 1.154-3.016, *p* = 0.011; HR = 2.014, 95%CI 1.196-3.391, *p* = 0.008, respectively), tumor stage (pTa/pT1 or pT2-4, HR = 3.359, 95%CI 1.860-6.066, *p* < 0.001; HR = 5.030, 95%CI 2.577-9.819, *p* < 0.001, respectively) and FA score (score 1: HR = 3.486, 95%CI 1.358-8.948, *p* = 0.009; HR = 3.485, 95%CI 1.363-8.913, *p* = 0.009, respectively; score 2: HR = 5.509, 95%CI 2.144-14.158, *p* < 0.001; HR = 5.521, 95%CI 2.074-14.697, *p* = 0.001, respectively) was an independent predictor for OS and CSS, but not tumor grade (G1-2 or G3, HR = 1.799, 95% CI, 0.940-3.368, *p* = 0.077; HR = 1.924, 95%CI 0.970-3.816, *p* = 0.061, respectively) (Table 3).

**Table 2:** Univariate Cox proportional hazard regression analyses of overall survival (OS) and cancer-specific survival (CSS) in 169 patients with UTUC

Variable	OS		CSS	
	HR (95% CI)	P value	HR (95% CI)	P value
Gender				
Male	1.000 (reference)	0.26	1.000 (reference)	0.400
Female	0.749 (0.453-1.239)		0.799 (0.473-1.349)	
Age (years)				
<65	1.000 (reference)	0.199	1.000 (reference)	0.091
≥65	1.369 (0.847-2.221)		1.564 (0.931-2.626)	
History of Smoking				
No	1.000 (reference)	0.521	1.000 (reference)	0.763
Yes	1.196 (0.692-2.069)		1.096 (0.604-1.989)	
Tumor Size				
<4cm	1.00 (reference)	0.036	1.000 (reference)	0.048
≥4cm	1.666 (1.033-2.685)		1.677 (1.004-2.765)	
Tumor Grade				
G1-2	1.000 (reference)	0.009	1.000 (reference)	0.009
G3	2.290 (1.230-4.263)		2.450 (1.246-4.820)	
Tumor Stage				
pTa/T1	1.00 (reference)	<0.001	1.000 (reference)	<0.001
pT2-4	3.807 (2.137-6.784)		4.795 (2.479-9.274)	
LVI				
Negative	1.00 (reference)	0.226	1.00 (reference)	0.223
Positive	1.391(0.815-2.376)		1.435(0.803-2.564)	
FA score				
0	1.000 (reference)		1.000 (reference)	
1	3.537 (1.380-9.064)	0.009	3.296 (1.290-8.419)	0.013
2	5.687 (2.219-14.574)	<0.001	4.104 (1.559-10.804)	0.004

OS = overall survival, CSS = cancer-specific survival, HR = hazard ratio, CI = confidence interval, LVI = Lymphovascular invasion, FA score = fibrinogen and albumin score

**Table 3:** Multivariate Cox proportional hazard regression analyses of overall survival (OS) and cancer-specific survival (CSS) in 169 patients with UTUC

Variable	OS		CSS	
	HR (95% CI)	*P* value	HR (95% CI)	*P* value
Tumor Size				
<4cm	1.000 (reference)	0.011	1.000 (reference)	0.008
≥4cm	1.865 (1.154-3.016)		2.014 (1.196-3.391)	
Tumor Grade				
G1-2	1.000 (reference)	0.077	1.000 (reference)	0.061
G3	1.779 (0.940-3.368)		1.924 (0.970-3.816)	
Tumor Stage				
pTa/T1	1.000 (reference)	<0.001	1.000 (reference)	<0.001
pT2-4	3.359 (1.860-6.066)		5.030 (2.577-9.819)	
FA score				
0	1.000 (reference)		1.000 (reference)	
1	3.486 (1.358-8.948)	0.009	3.485 (1.363-8.913)	0.009
2	5.509 (2.144-14.158)	<0.001	5.521 (2.074-14.697)	0.001

OS = overall survival, CSS = cancer-specific survival, HR = hazard ratio, CI = confidence interval, FA score = fibrinogen and albumin score

**Figure 2:**
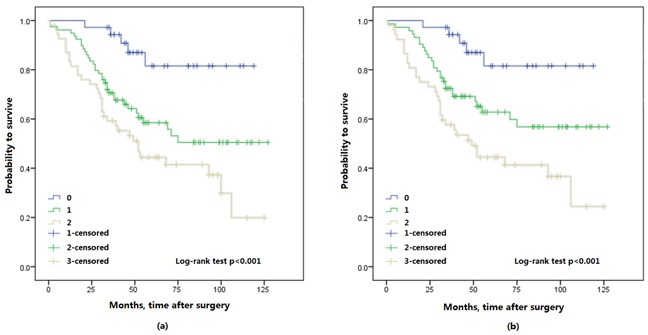
Kaplan-Meier survival curves of all patients with upper urinary tract urothelial carcinoma stratified by FA score, a. overall survival (OS); b. cancer specific survival

**Figure 3:**
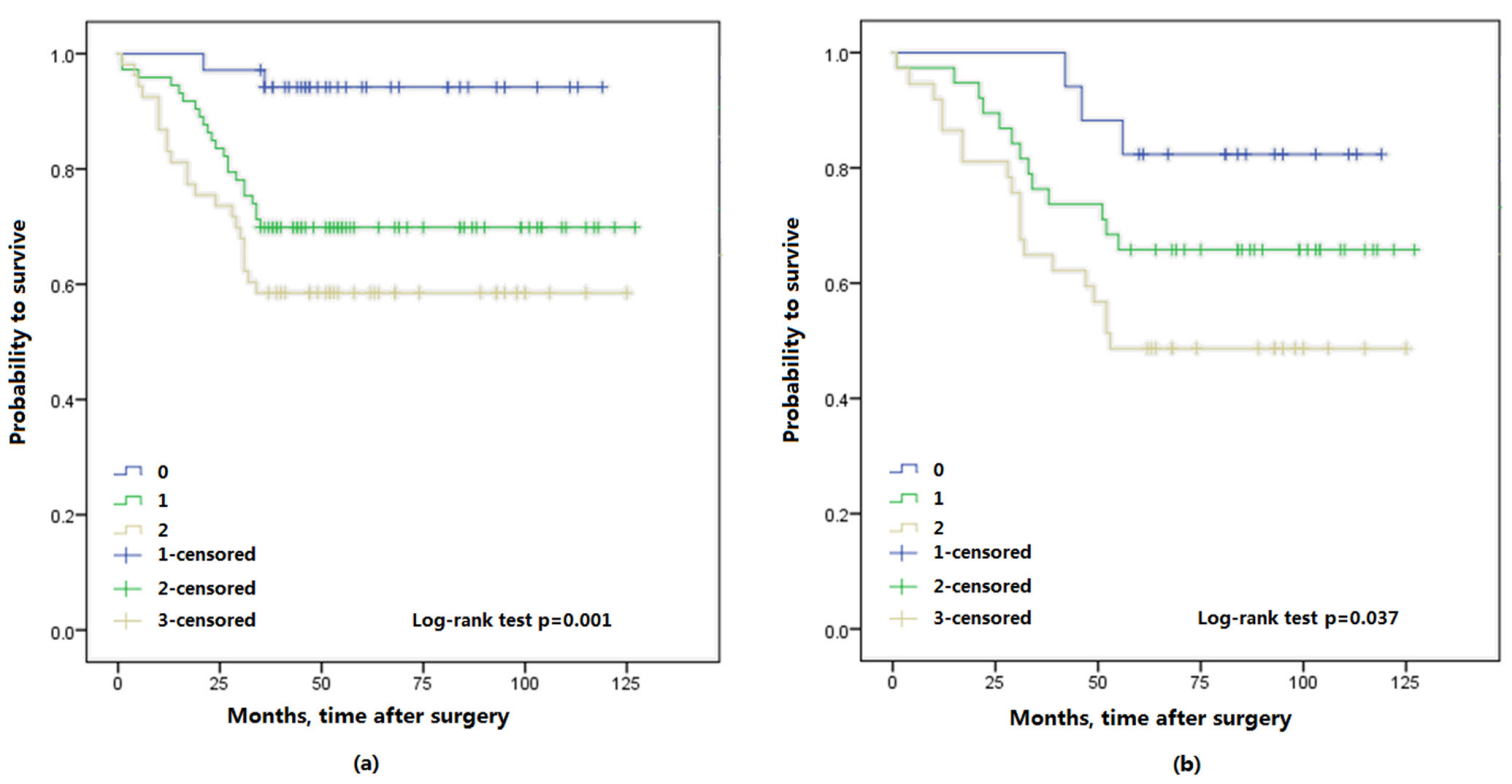
Kaplan-Meier survival curves for 3-year and 5-year overall survival (OS) of 161 and 92 patients with upper urinary tract urothelial carcinoma stratified by FA score, respectively, a. 3-OS; b. 5-OS

The Kaplan-Meier survival analysis showed that preoperative FA score was significantly associated with OS and CSS, a higher FA score in patients with shorter OS (*p* < 0.001) and CSS (*p* < 0.001) (Figure 2). Furthermore, 3- and 5-year OS were also identified in this study, 3-year OS was 94.3% (33/35) in FA score 0, 69.9% (51/73) in FA score 1 and 58.5% (31/53) in FA score 2. 5-year OS was 82.4% (14/17) in FA score 0, and 65.8% (25/38) in FA score 1, 48.6% (18/37) in FA score 2. Thus, FA score could predicted significantly poorer OS (*p* = 0.001; *p* = 0.037; respectively) (Figure 3).

**Figure 4:**
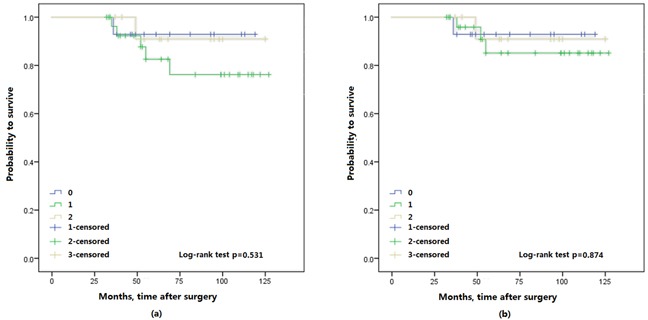
Kaplan-Meier survival curves of Ta/T1 patients with upper urinary tract urothelial carcinoma stratified by FA score, a. overall survival (OS); b. cancer specific survival

**Figure 5:**
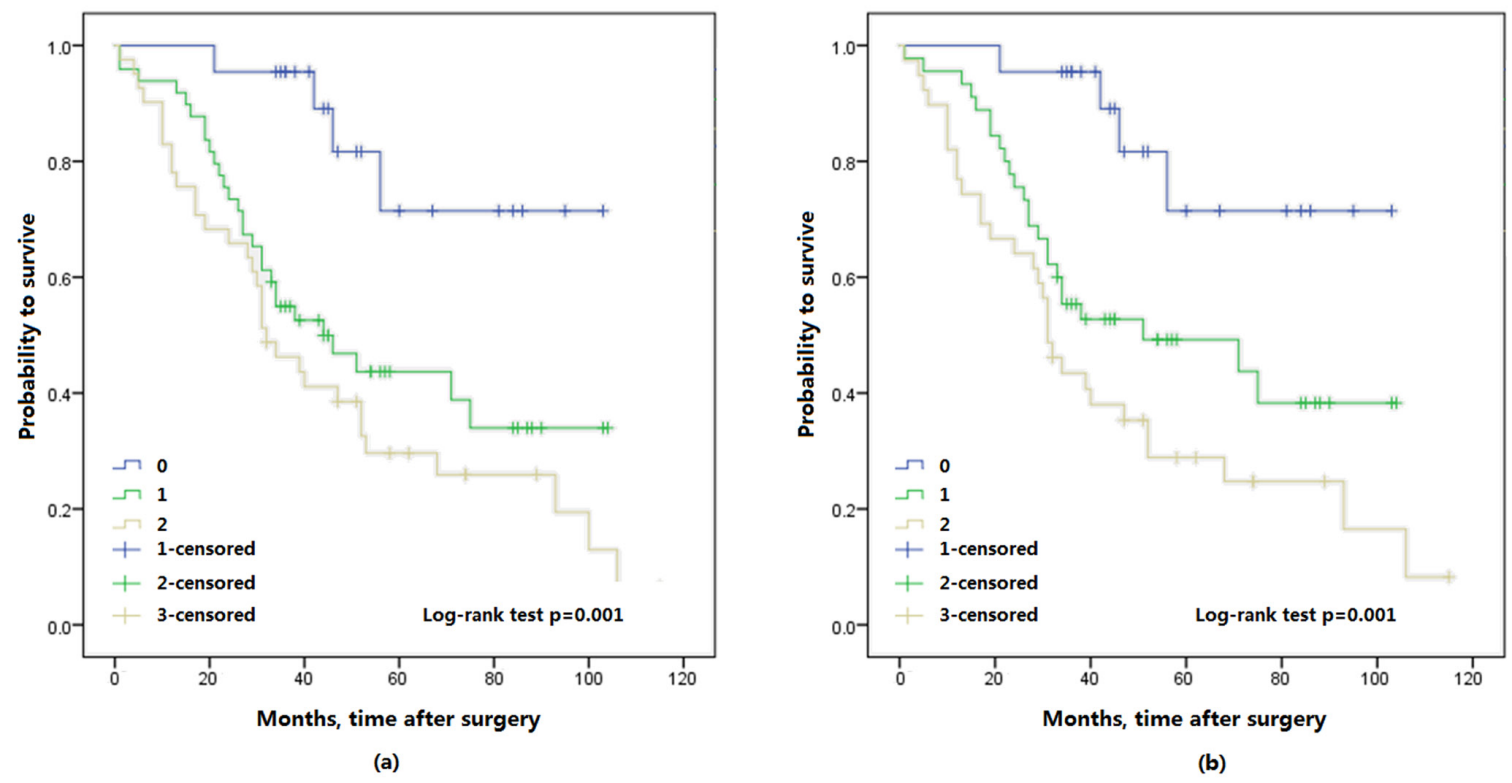
Kaplan-Meier survival curves of T2-4 patients with upper urinary tract urothelial carcinoma stratified by FA score, a. overall survival (OS); b. cancer specific survival

In addition, we further evaluated whether the different tumor pathological stages were associated with the OS. No difference was found in patients with pTa/T1 stage for both OS (*p* = 0.513) and CSS (*p* = 0.874) (Figure 4), but in patients with pT2-4 stage for both OS (*p* = 0.001) and CSS (*p* = 0.001) (Figure 5).

## DISCUSSION

Due to lack of specific biomarkers for predicting survival of patient with UTUC, the stratification of prognostic risk mainly depended on pathological stage, tumor grade and lymph node metastasis. Now increasing evidence of hemostatic factors and SIR showed they might be heavily involved in the development and progress of various human cancers [9, 13, 16]. In the previous studies [6–9, 17], fibrinogen, albumin, neutrophil to lymphocyte ratio and some other hematology indexes were reported which could predict the prognosis of patients with UTUC. Tanaka et al [9] indicated that the strongly predicted value of fibrinogen was 4.50g/L, Pichler et al [6] reported 3.7g/L and Zhang et al [14] showed 3.54g/L, we set the cutoff value of 3.53g/L by ROC curve analysis. Ku et al [8] showed albumin of 35g/L was an independent factor to evaluate the prognostic value in patients with UTUC. Therefore, it seemed reasonable to use the combination of plasma fibrinogen and serum albumin for predicting survival of patients, and it could be more convincible because of the integration of hemostatic factors, malnutrition and SIR.

Elevated plasma fibrinogen is usually caused by non-malignant factors, such as myocardial infarction, acute infection, et al. Meanwhile, more evidence shows fibrinogen plays an important role on development and progression of cancer. Act as a molecular bridge, fibrinogen could promote stable adhesion among tumor cells, platelets and endothelial cells [18–21]. Palumbo et al [22] reported fibrinogen was a critical determinant of the metastatic potential of circulating tumor cells and facilitated tumor formation and dissemination. Furthermore, another potential mechanism of influencing prognostic was that fibrinogen could enhance tumor migration by impeding natural killer or other cytotoxic cells elimination of tumor cells [23, 24]. Moreover, plasma fibrinogen takes part in the coagulation cascade and elevated fibrinogen might induce thromboembolism events. However, it still remains controversial whether higher plasma fibrinogen has constantly relationship with higher risk of thromboembolism. Man et al [25] reported high plasma fibrinogen were associated with high risk of venous thromboembolism (VTE) in patients with epithelial ovarian cancer, but Tiedje et al [26] revealed no significant relationship was found between VTE and fibrinogen.

Serum albumin is produced by the liver and helps to maintain intravascular oncotic pressure, facilitate transport of substances and acts as a free radical scavenger [16]. Meanwhile, albumin is one of the most widely used markers for evaluating nutritional status. Malnutrition which might be reflected by serum albumin level could weaken the human defense mechanisms, including anatomic barriers, cellular and humoral immunity, and phagocyte function [13]. Several studies have reported hypoalbuminemia is associated with poor prognosis and post-operative complications in patients with various cancers [8, 13, 27]. However, Ataseven et al [13] suggested that no correlation between hypoalbuminemia and body mass index (BMI) which was an index of nutritional status in patients, but could reflect prognosis. More evidence has shown that hypoalbuminemia not only reflects patient’s malnutrition and directly reveals prognosis of survival, but also is considered a status of inflammatory response [8].

The chronic inflammatory response is associated with proliferation, development, progression and metastasis of tumor cells, as well as angiogenesis. Hypoalbuminemia may be caused by cytokines which is released by the tumor cell, such as interleukin(IL)-6, which blocks hepatocyte albumin production [28]. Tumor necrosis factor(TNF)-α has relationship with albumin levels, thus, TNF-α and cytokine levels may be a surrogate for more aggressive disease [29].

The present study shows the preoperative FA score is significantly associated with OS and CSS. Nevertheless, the results should be interpreted with caution. The potentially confounding factors related to prognosis in patients with UTUC had not calculated HR and 95%CI in the present study because of the limited number, such as tumor focality, ECOG-PS, operation approach and adjuvant therapy, this could be a potential bias.

The advantages for using the FA score are its low cost, routine measured and easy accessibility in clinical practice. And as a retrospective study, the limitations of the present study are inherent to the design. Moreover, single-institution and limited patients are another potential selection bias for the present study. Furthermore, part of patients in the present study had shortage of follow-up time. Only 92 patients were involved in 5-OS analysis. However, even considering these limitations, the present study suggests the FA score as a potential independent prognostic factor for OS and CSS in patients with UTUC. A well-designed, prospective study with multicenter involvement and a larger number of patients is needed.

## MATERIALS AND METHODS

### Patients

This retrospective analysis included clinicopathologic and follow-up data from 169 patients with non-metastatic UTUC who received RNU or segmental resection at the department of urology at the Qilu hospital of Shandong University from January 2006 to December 2013. Patients with following condition were excluded from this study: (1) No data on preoperative plasma fibrinogen and serum albumin level; (2) Patients with autoimmune disease, cancer in other system or received neo-adjuvant chemotherapy and radiotherapy; (3) Pathological type was not urothelial carcinoma; (4) Distant metastasis was diagnosed; (5) No data on patients’ follow-up. This study was approved by the Institutional Ethics Committee of the Qilu Hospital of Shandong University.

All patients underwent routine hematologic examination, computerized tomography/magnetic resonance imaging, cystoscopy, urinary cytology and/or ureteroscopy with tissue biopsy to diagnose patients with localized non-metastatic UTUC before surgery. Segmental resection was conducted in imperative cases, such as chronic renal insufficiency, impaired renal function, solitary kidney, parenchymal rarefication of the contralateral kidney or American Society of Anesthesiologists score 4. Pathological T stages were uniformly adjusted according to the 2009 TNM classification of UTUC and tumor grade was assessed based on the World Health Organization (WHO) 1973 guidelines. LVI was defined as the presence of tumor cells within a lymph or vein duct. Preoperative baseline clinicopathologic and laboratory data, such as age, gender history of smoking, history of bladder cancer, tumor size, location and pathological type were reviewed and obtained from the electronic medical records. The tumor size was defined as the longest diameter of the general post-operative pathological specimens. ECOG-PS was conducted to evaluate the condition of patients before surgery.

### Follow-up and treatment

All patients were regularly followed with physical examination, urine routines routine blood test, biochemical tests and cystoscopy every 3 months for the first year, every 6 months in the 2-3 years and yearly thereafter for more than 3 years, computerized tomography urography or magnetic resonance imaging every 6 months for the 1-2 years, yearly thereafter for more than 2 years. Adjuvant treatments which included bladder instillation of chemotherapy, systemic chemotherapy and radiotherapy were planned according to the tumor stage, doctor’s selection and patient’s desire. OS was defined as the time from surgery to death from all causes. Cancer-specific survival (CSS) was defined as the time from surgery to cancer related death.

### Statistical analysis

Statistical analysis was performed using the Statistical Package for Social Science (SPSS for Windows, version 23.0, SPSS Inc., Chicago, IL) program. The fibrinogen and albumin level were shown as the mean and SD. Correlations between categorical variables were evaluated by the Pearson’s chi square test or Fisher’s exact test in this study. The probable cutoff value level for the fibrinogen and albumin level was determined by applying receiver operating curve (ROC) analysis. In short, the optimal cutoff value had the best sensitivity and specificity values in this study. And the most optimal cutoff value was used to further analysis. In OS and CSS analyses, the Kaplan-Meier method was used to evaluate the survival rates in different groups and the equivalences of the survival curves were tested by log-rank test. Besides, the Cox proportional hazards regression model was applied in univariate and multivariate analyses. The two-sided p value was used in our analyses, and a p value of less than 0.05 considered statistically significant.

## CONCLUSIONS

The present study shows the evaluation of preoperative FA score can be regarded as an independent prognostic factor for predicting OS and CSS of patients with UTUC. The fibrinogen and albumin levels are low cost, routine measured and easy accessibility in clinical practice. Thus, the combination of plasma fibrinogen and serum albumin levels should be involved in the prognostic indicators, and improve the personalized multidisciplinary therapy for patients with UTUC. However, the detailed mechanisms of FA score in UTUC should be explained further.

## Abbreviations

UTUC: upper urinary tract urothelial carcinoma
OS: overall survival
CSS: cancer specific survival
UC: urothelial carcinoma
BCa: bladder cancer
SIR: systemic inflammatory response
SD: standard deviation
LVI: Lymphovascular invasion
RNU: radical nephroureterectomy
VTE: venous thromboembolism
TNF: tumor necrosis factor
IL: interleukin
WHO: World Health Organization
ECOG-PS: Eastern Cooperative Oncology Group performance status
SPSS: Statistical Package for Social Science
ROC: receiver operating curve.
